# Comparison of Platelet-Rich Plasma Properties Used in Athletic Musculoskeletal Conditions: A Clinical Practice Report

**DOI:** 10.7759/cureus.84080

**Published:** 2025-05-14

**Authors:** António Pais Neto, Tiago B Cunha, Nuno Pereira, Miguel Reis e Silva

**Affiliations:** 1 Physical Medicine and Rehabilitation, Centro de Medicina de Reabilitação de Alcoitão, Cascais, PRT; 2 Physical Medicine and Rehabilitation, Health and Performance, Sport Lisboa e Benfica, Lisboa, PRT

**Keywords:** athletes, musculoskeletal injuries, platelet concentration, platelet-rich plasma (prp), sports-related injuries

## Abstract

This study evaluates the properties of platelet-rich plasma (PRP) preparations used by a professional sports club's medical department for treating musculoskeletal conditions in athletes. PRP samples were prepared using 20 ml and 50 ml Hy-tissue® PRP Kits (Fidia Farmaceutici S.p.A., Abano Terme, Italy) from 13 total blood samples. They were analyzed to assess platelet (PLT), leukocyte, and red blood cell concentrations. Results showed both kits effectively concentrated PLT with an average enrichment factor of 2.9×, though the 50 ml kit produced higher total PLT counts (4.7 Bl) than the 20 ml kit (1.7 Bl). White and red blood cell levels were significantly reduced, indicating high PRP purity. DEPA (dose of injected PLT, efficiency of production, purity of the PRP, activation of the PRP) classification revealed a superior quality score, from A to D in the three prior parameters, for the 50 ml kit (BCA vs. CCA). Despite acceptable PLT concentration, the efficiency of PLT recovery was low across both kits. The findings underscore the importance of evaluating PRP characteristics to optimize therapeutic outcomes and highlight the need for protocol refinement to enhance preparation efficiency. These results advocate for standardized reporting and classification to support evidence-based application of PRP in sports medicine.

## Introduction

Platelet-rich plasma (PRP) has emerged as a promising therapeutic approach for treating musculoskeletal conditions, particularly in athletes who experience frequent injuries [1]. PRP is a concentration of platelets (PLT) derived from the patient's blood that may play a crucial role in tissue repair and regeneration [2].

The therapeutic benefits of PRP can be attributed to its rich composition of bioactive molecules, including growth factors such as PLT-derived growth factor, transforming growth factor-beta, and vascular endothelial growth factor, which stimulate cell proliferation, angiogenesis, and collagen synthesis [3]. By promoting tissue repair and modulating inflammatory responses, PRP aims to enhance recovery time and return athletes to peak performance [4] by accelerating healing processes, reducing pain, and improving functional recovery after musculoskeletal injuries such as tendinopathies, ligament sprains, and cartilage damage [5,6]. However, no research strongly advocates using PRP compared with traditional management strategies [7].

Clinicians often do not fully understand the impact of PRP characteristics and efficacy. Unfortunately, many published research articles do not include details on PRP properties or the volume administered [2,8]. The assessment of PRP properties is vital to ensure its efficacy in clinical practice, including the concentration of PLT and leukocytes and the method of preparation [9], which can significantly influence the outcomes of PRP treatment. Establishing a standardized reporting system for PRP is crucial to improve communication among clinicians and enhance the interpretation and synthesis of scientific studies [2]. The DEPA (dose of injected PLT, efficiency of production, purity of the PRP, activation of the PRP) classification provides a comprehensive framework for standardizing PRP preparations, aiming to improve consistency and predictability in clinical applications [10].

Thus, understanding the specific properties of PRP is crucial to optimizing its application in managing musculoskeletal injuries and tailoring treatments to individual patient needs. This study presents the current practice of a sports club medical department using PRP to treat musculoskeletal conditions. According to recent research and best practice guidelines, it analyzes and classifies its properties.

## Materials and methods

Reports of blood analysis, including volume and cell concentration from blood samples and PRP preparations using a sterile, single-use medical device, the Hy-tissue® PRP Kit (Fidia Farmaceutici S.p.A., Abano Terme, Italy), designed to extract and process either 20 ml or 50 ml of anticoagulated blood to obtain PRP, were collected from an automated cell counter, the Porteal BioQControl®. The Duodrafter II® centrifuge (Fidia Farmaceutici S.p.A., Abano Terme, Italy) was used with standard programmed settings (1800 revolutions per minute and seven minutes). Data was gathered from three consecutive months (September to November of 2020) of PRP samples used in treating musculoskeletal conditions by Clinica Benfica - SL Benfica Medical Department, where the study was conducted. All samples were available during that period, and the 20 ml and 50 ml kits were included. Samples with missing data regarding cell concentration or the type of kit used for PRP preparation were excluded from analysis. The parameters extracted from the automated cell counter included PLT, red blood cell (RBC), and white blood cell (WBC) concentrations in blood (B) and PRP. The PLT concentration ratio (PLT PRP/B), along with the total PLT count in billions (Bl) within PRP and the PLT count per milliliter (PLT/ml) in PRP, were calculated.

A descriptive analysis and a literature review were conducted to classify the study sample’s PRP properties according to the most recent reports on the optimal PRP characteristics for achieving the best clinical results.

## Results

**Figure 1:**
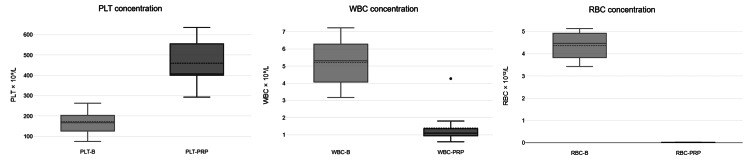
Blood cell concentration in blood and PRP samples PRP: platelet-rich plasma, PLT-B: platelet concentration in whole blood (baseline), PLT-PRP: platelet concentration in platelet-rich plasma, WBC-B: white blood cell concentration in whole blood (baseline), WBC-PRP: white blood cell concentration in platelet-rich plasma, RBC-B: red blood cell concentration in whole blood (baseline), RBC-PRP: red blood cell concentration in platelet-rich plasma

PRP properties were analyzed on blood samples from 10 male and three female athletes. The mean age was 24 years, ranging from 16 to 37 years. The 50 ml kit was used for 10 athletes, yielding 10 ml of PRP, and the 20 ml kit was used for three athletes, yielding 4 ml of PRP. Neither sample was activated prior to the PRP injection. Variations in PLT concentrations were observed across individual blood samples. The median PLT concentration was 167 × 10⁹/L, with an interquartile range of 124-215.5 × 10⁹/L (minimum: 75 × 10⁹/L and maximum: 263 × 10⁹/L) (Figure 1). The results demonstrate that both kits effectively concentrated PLT in the PRP fraction, with an average PLT PRP/B of 2.9 ± 0.98. This indicates that the PRP contained approximately three times the PLT concentration of the blood sample, on average.

**Figure 2:**
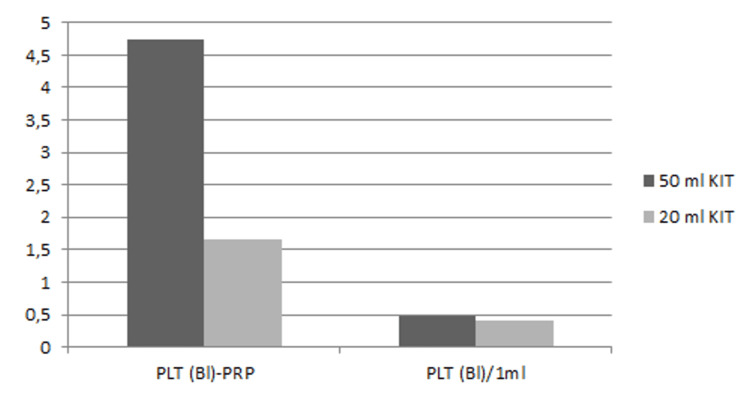
Total PLT count in PRP and PLT count per ml in both 50 ml and 20 ml kits PLT (Bl)-PRP: number of platelets in billions per platelet-rich plasma, PLT (Bl)/1 ml: number of platelets in billions per milliliter

The total PLT count in PRP varied depending on the kit used. The 20 ml kit produced 4 ml of PRP per sample, resulting in an average PLT count of 1.7 ± 0.53 Bl. In contrast, the 50 ml kit, yielding 10 ml of PRP per sample, showed a higher PLT count of 4.7 ± 1.5 Bl. While both kits enhanced PLT concentration similarly, the absolute number of PLT in the final PRP product was higher in the 50 ml kit, as expected due to the greater blood volume processed. When evaluating PLT/1 ml in PRP samples, the overall mean was 0.46 ± 0.11 Bl/ml (Figure 2).

The WBC concentration in whole blood samples (WBC-B) ranged between 3.17 × 10⁹/L and 7.23 × 10⁹/L, with an average value of 4.92 × 10⁹/L. After PRP processing, the WBC concentration in PRP (WBC-PRP) varied between 0.78 × 10⁹/L and 4.27 × 10⁹/L, with an average value of 1.39 × 10⁹/L, with no significant difference between kits. This indicates a significant reduction in WBCs (Figure 2).

The RBC concentration in whole blood samples (RBC-B) ranged from 3.43 × 10¹²/L to 5.13 × 10¹²/L, with an average value of 4.37 × 10¹²/L. Following PRP preparation, the RBC concentration in PRP (RBC-PRP) was substantially reduced across all samples, ranging from 0.01 × 10¹²/L to 0.03 × 10¹²/L, with no significant difference between kits. This indicates effective RBC depletion in the final PRP product (Figure 2).

The DEPA classification system evaluates PRP based on the dose of PLT (D), efficiency of PLT recovery (E), purity of PRP (P), and activation (A) using grades A, B, C, and D, and each DEPA component is graded independently on an A-D scale. The same letters are reused for each parameter and indicate an aggregate performance rating. Since none of the PRP samples in this study were activated before injection, the activation criterion (A) is not applicable. The PRP samples were classified according to the first three criteria (Table 1).

**Table 1:** PRP properties and classification according to DEPA classification Dose of injected PLT (billions): A: >5 (very high dose), B: 3–5 (high dose), C: 1-3 (medium dose), D: <1 (low dose) Efficiency of the process (PLT recovery rate %): A: >90% (high), B: 70-90% (medium), C: 30-70% (low), D: <30% (poor) Purity of PRP (relative composition in PLT %): A: >90% (very pure PRP), B: 70-90% (pure PRP), C: 30-70% (heterogeneous PRP), D: <30% (whole blood PRP) PLT: platelet, PRP: platelet-rich plasma, DEPA: dose of platelets, efficiency of platelet recovery, purity of PRP, and activation

	PRP volume	Dose of injected PLT (billions)	Efficiency of the process (PLT recovery rate %)	Purity of the PRP (relative composition in PLT %)	Final DEPA score
50 ml kit	10 ml	4.7	59.1 ± 21.1	96.4 ± 1.6	BCA
20 ml kit	4 ml	1.7	51.2 ± 14.7	95.2 ± 1.1	CCA

Regarding the 50 ml kit, the total dose of PLT per PRP sample averaged 4.7 Bl PLT, with individual values ranging from 3.4 to 6.4 Bl, indicating a high to very high PLT dose suitable for clinical applications. The PLT recovery efficiency, representing the percentage of PLT retained in PRP relative to blood, had an average value of 59.1%, indicating that the PRP preparation process resulted in low efficiency in PLT recovery. Purity is the proportion of PLT relative to other blood components, particularly RBCs and WBCs. The samples demonstrated a high purity level, with an average purity of 96.4%, indicating that most unwanted cellular components were successfully removed, representing a very pure PRP with minimal contamination from other blood cells.

On the other hand, the average PLT count across the samples from the 20 ml kit was 1.7 Bl PLT, with individual values ranging from 1.2 to 2.2 Bl, indicating a medium PLT dose. The PLT recovery efficiency had an average value of 51.2%, meaning that the PRP preparation process achieved a low level of PLT recovery. The average purity in this dataset was 95.2%, indicating that most unwanted cellular components were successfully eliminated, representing high-purity PRP.

## Discussion

The results indicate that both PRP preparation kits successfully enhance PLT concentration from whole blood, with a similar PLT PRP/B ratio across samples. The findings confirm the effectiveness of both PRP kits in concentrating PLT, achieving an average increase of 2.9×. Therefore, the choice between kits may depend on the desired PRP volume and total PLT count.

A previous in vitro study has shown that PRP with higher PLT concentrations releases greater amounts of growth factors, as seen in the comparison of 4.69× vs. 1.99× PLT concentration [11]. A recent study found that higher PLT concentration in PRP correlated with greater clinical improvement and lower failure rates over a 12-month follow-up period in patients with osteoarthritis (OA) [12]; however, there is limited evidence regarding the optimal PRP concentration for other musculoskeletal conditions.

The 50 ml kit yielded a higher absolute PLT count (4.7 BI), making it preferable in scenarios where a larger PLT dose is desirable. Conversely, the 20 ml kit produced a smaller PRP volume and PLT count (1.7 Bl), which may be more suitable for localized treatments or cases requiring a lower PRP volume (i.e., intratendinous requirements). However, the lower PLT dose appears to be less advantageous.

A recent systematic review and meta-analysis examined the impact of PLT dosage in PRP injections for musculoskeletal conditions and identified a potential dose-response relationship. The study suggested that a total PLT count exceeding 10 Bl may improve clinical outcomes, particularly in knee OA. However, the same study noted that a lower dose might be sufficient, although current literature remains inconclusive on this matter [13].

In the RESTORE trial, participants received intra-articular injections of 5 mL of PRP into the knee joint with a medium PLT dose of 1.6 Bl. This study concluded that PRP injections did not provide significant benefits over a saline placebo in improving symptoms or altering joint structure in patients with knee OA over 12 months [14]. On the other hand, Chu et al. found that PRP was superior to saline placebo in treating knee OA, providing effective symptom relief for at least 24 months and slowing disease progression with a 4.1 Bl PRP dose [15].

These findings suggest that total PLT count is a critical factor directly related to therapeutic potential. However, concentration and volume also appear to matter, especially in ensuring good growth factor delivery. Additionally, the observed PLT/1 ml values provide insights into the PLT density of the final PRP product in our practice, offering relevant clinical data for future research involving PLT dosage per 1 ml injected.

Regarding PRP efficiency, some studies explore optimization strategies to enhance PLT recovery and minimize variability across blood samples. The double centrifugation technique is commonly used to concentrate PLT [16] effectively, and the choice of anticoagulant can influence PRP quality. Research comparing sodium citrate (SC) and ethylenediaminetetraacetic acid (EDTA) as anticoagulants has not agreed on the most effective option. Studies have reported conflicting results regarding PLT recovery rates and growth factor levels. However, a recent study found that EDTA provided superior PLT recovery while maintaining PLT functionality during PRP preparation [17]. Our current practice has room for improvement, as the process efficiency was low. This may be related to our protocol, which includes only one centrifugation step and uses SC as the anticoagulant.

On the other hand, our results revealed an ideal reduction in RBC content in PRP. This outcome is desirable, as excessive RBCs in PRP can contribute to oxidative stress, increased inflammation, and discomfort upon injection into tissues [18].

Regarding WBC content, our clinical practice prioritizes low leukocyte concentration in PRP. Several studies have compared leukocyte-rich PRP (LR-PRP) and leukocyte-poor PRP (LP-PRP) in the treatment of musculoskeletal conditions, particularly knee OA. However, no significant differences were found between LP-PRP and LR-PRP concerning patient-reported outcomes [19,20]. Similar results were observed following rotator cuff repair surgery [21].

Research has shown that leukocyte concentration in PRP influences the levels of growth factors and proteases. Specifically, higher leukocyte concentration correlates with increased levels of specific growth factors and matrix metalloproteinase-9, which is associated with tissue degradation and catabolic properties [22] that may stimulate and kick-start tissue regeneration. While both LR-PRP and LP-PRP have demonstrated efficacy, LP-PRP may reduce adverse reactions [23] and improve specific clinical outcomes. Thus, the choice between LR-PRP and LP-PRP should be tailored to the specific musculoskeletal condition, supporting individualized PRP treatment.

Regarding PRP exogenous activation, additives such as calcium chloride and thrombin can promote the degranulation of PLT, leading to the immediate and rapid release of growth factors [24]. A recent systematic review and meta-analysis showed that activated PRP was more effective than non-activated PRP in reducing pain and improving function in patients with knee OA [25], although there is no strong evidence that fast cytokine release improves outcomes in other musculoskeletal conditions, and results remain contradictory [26]. For this reason, non-activated PRP remains our standard practice.

Although numerous efforts have been made to characterize and classify PRP, a clear consensus and universally accepted classification system are still lacking [27]. The DEPA classification addresses four key PRP properties and preparation aspects, providing valuable insights into PRP quality. It emphasizes evaluating multiple quantitative and qualitative parameters to ensure consistency and efficacy in PRP-based clinical applications.

The final DEPA scores for our sample were BCA for the 50 ml kit and CCA for the 20 ml kit, reflecting the former's superior performance, primarily due to its higher PLT count. We believe that improving the efficiency of both kits could increase the PLT dose and, consequently, their overall DEPA score.

Some limitations of this study can be noted, including the small sample size; the lack of information regarding the medical conditions or injuries treated with PRP injections; the criteria used to select the kit for each case; and the absence of reported clinical outcomes due to restricted access to athletes’ medical records, although assessing clinical outcomes was not the primary focus of this study.

## Conclusions

This study highlights the importance of conducting a critical self-assessment of the characteristics of the PRP used in treating musculoskeletal pathologies in athletes to improve clinical practice and enhance patient outcomes. In the future, efforts should focus on identifying strategies to increase the efficiency of PRP preparation within our clinical setting and designing studies that systematically report clinical outcomes.
